# Uncovering the genetic basis for quality traits in the Mediterranean old wheat germplasm and phenotypic and genomic prediction assessment by cross-validation test

**DOI:** 10.3389/fpls.2023.1127357

**Published:** 2023-01-27

**Authors:** Venkata Rami Reddy Yannam, Marta Lopes, Carlos Guzman, Jose Miguel Soriano

**Affiliations:** ^1^ Sustainable Field Crops Program, Institute for Food and Agricultural Research and Technology (IRTA), Lleida, Spain; ^2^ Departamento de Genética, Escuela Técnica Superior de Ingeniería Agronómica y de Montes, Universidad de Córdoba, Córdoba, Spain

**Keywords:** *Triticum aestivum*, genome-wide association study, loaf volume prediction, quantitative trait locus, candidate genes, wheat quality

## Abstract

The release of new wheat varieties is based on two main characteristics, grain yield and quality, to meet the consumer’s demand. Identifying the genetic architecture for yield and key quality traits has wide attention for genetic improvement to meet the global requirement. In this sense, the use of landraces represents an impressive source of natural allelic variation. In this study, a genome-wide association analysis (GWAS) with PCA and kinship matrix was performed to detect QTLs in bread wheat for fifteen quality and agronomic traits using 170 diverse landraces from 24 Mediterranean countries in two years of field trials. A total of 53 QTL hotspots containing 165 significant marker-trait associations (MTAs) were located across the genome for quality and agronomical traits except for chromosome 2D. The major specific QTL hotspots for quality traits were QTL_3B.3 (13 MTAs with a mean PVE of 8.2%) and QTL_4A.3 (15 MTAs, mean PVE of 11.0%), and for yield-related traits were QTL_2B.1 (8 MTAs, mean PVE of 7.4%) and QTL_4B.2 (5 MTAs, mean PVE of 10.0%). A search for candidate genes (CG) identified 807 gene models within the QTL hotspots. Ten of these CGs were expressed specifically in grain supporting the role of identified QTLs in Landraces, associated to bread wheat quality traits and grain formation. A cross-validation approach within the collection was performed to calculate the accuracies of genomic prediction for quality and agronomical traits, ranging from -0.03 to 0.64 for quality and 0.46 to 0.65 for agronomic traits. In addition, five prediction equations using the phenotypic data were developed to predict bread loaf volume in landraces. The prediction ability varied from 0.67 to 0.82 depending on the complexity of the traits considered to predict loaf volume.

## Introduction

Wheat (*Triticum aestivum* L.) is one of the globally essential cereals cultivated in a wide range of latitudes and is usually consumed in baked products. Moreover, wheat contributes 18% of the daily intake of calories and 20% of protein. To ensure global food security in the future, overall wheat production needs to increase but producing high nutritional value food as society demands (Battenfield et al., 2016). Assessing the grain quality traits is a bottleneck for the grain value chain in the current scenario. Additionally, increasing yield without a negative effect on grain quality is a puzzle for breeders because an increase in yield typically leads to a decrease in protein content, which often reduces processing and end-use quality attributes. Identifying the genetic architecture for quality traits and maintaining high yields is vital for the breeders to develop new varieties depending on end-use products. The bread wheat is classified by the end-use baked products. The different products obtained from bread wheat, like noodles, cookies, pastries, and leavened and unleavened bread, require specific characteristics such as flour quality, protein concentration, grain hardness, and gluten strength (Pena, 2002; Guzmán et al., 2015).

The properties considered by the millers are mainly kernel weight, hardness, colour, grain protein, and test weight (weight per volume) which are related to grain yield and quality with varying heritability (Aydin et al., 2010; Guzmán et al., 2016; Irfan Ullah et al., 2021). Grain hardness is another essential characteristic for millers. This trait can be explained based on how strongly the starch granules are linked to the protein matrix. The harder the grain is, the more energy is required during the milling process, resulting in more starch damage than soft grain (Giroux and Morris, 1997). The water absorption by dough is higher in the hard grain due to the higher intensity of starch damage which is not preferable for pastries and cookies. In addition to the absorption of water, the higher starch damage leads to the hydrolysis of starch into fermented sugars leading to higher loaf volume (Pomeranz and Williams, 1990). Among the quality parameters, protein content (in grain and flour) is the primary conventional indicator of nutritional value (Zhao et al., 2010). Among the different types of proteins in the grain, gliadins and glutenins are the main storage proteins and components. Their compositions and proportions are responsible for the baking quality, dough strength, and wheat’s viscoelastic properties (Torbica et al., 2007). The dough’s rheological and physiochemical properties consist of viscosity or stiffness, tenacity (P) (maximum energy spent to deform the dough), strength (alveograph, W) is the persistence of viscosity upon extension, extensibility (L) which is expressed as the distance of the dough extends without breaking, tenacity/extensibility (P/L), and dough mixing time (MT) of the dough decide the end-use product (Pena, 2002).

During its domestication, wheat was selected mainly for adaptation to the local environment by considering traits like flowering time, biotic and abiotic stress resistance, yield parameters, and plant height (Royo et al., 2017). Selection intensity during the green revolution was especially for yield by selecting the semi-dwarf plants. However, landraces are an important source of maintaining biodiversity, and although their low yield in comparison with the modern improved cultivars, nowadays, the interest in landraces by many breeders and farmers is increasing because of their nutritional values and sensory properties (Casañas et al., 2017). Usually, the landraces have higher grain protein with poor rheological properties compared to modern cultivars (Guzman et al., 2019). Knowing the desired untapped gene pool and allelic variation in landraces to improve the quality traits in bread wheat is a vital strategy to overcome the challenges faced by breeders to meet the global requirements of nutritional food in consideration of climatic change and the rapid growth of the human population in addition to meet the requirements of the millers and industrial bakers in manufacturing baking products (Lopes et al., 2015). Quantitative trait loci (QTL) have been mapped in Iranian and Spanish bread wheat landraces for end-use quality traits using genome-wide association studies (GWAS) (Abdipour et al., 2016; López-Fernández et al., 2021; PU et al., 2022; Rabieyan et al., 2022). Additionally, it has been documented that the Mediterranean durum wheat landraces exhibit a broad genetic variation for quality and yield traits (Moragues et al., 2006; Nazco et al., 2014; Lopes et al., 2015; Roselló et al., 2018; Roselló et al., 2019). Therefore, association studies in Mediterranean bread wheat landraces give a new non-improved allelic source and allow targeted introduction into elite material.

The fundamental focus of this study is to study the genetic architecture and candidate genes associated with the quality and grain yield traits in a collection of 170 Mediterranean bread wheat landraces through a GWAS approach. In addition to detecting the QTLs associated with the targeted characteristics, we also evaluate the genomic selection prediction accuracies and prediction of loaf volume with different quality parameters, which serve as the pipeline for breeders to select the individuals in the early stages and allow the breeders to evaluate a large population.

## Material and methods

### Plant material

The plant material consisted of 170 bread wheat (*Triticum aestivum L.*) landraces from 24 Mediterranean countries derived from the MED6WHEAT IRTA panel (Rufo et al., 2019) (**Supplementary Table 1**) and is structured into three genetic sub-populations (SPs) (Rufo et al., 2019): SP1, west Mediterranean landraces (43 accessions); SP2, north Mediterranean landraces (59 accessions); SP3, east Mediterranean landraces (42 accessions); and 26 were considered as admixed genotypes.

### Field trials

The panel was evaluated during two growing seasons (2016 and 2017) in rainfed conditions at Gimenells, Lleida (Northeast Spain). The experimental design followed a non-replicated augmented design using six genotypes, and two replicated checks per line, the cultivars ‘Anza’ and ‘Soissons’. Each experimental plot was 3m x 1.2m with 250 germinable seeds per m^-2^.

Grain yield (GY, t/ha^-1^) was determined by mechanically harvesting the plots at ripening and is expressed at a 12% moisture level. Thousand kernel weight (TKW, g) was determined by counting the grains in 10 g drawn randomly from harvested grains of each plot. Duration of grain filling (GFD, days) was calculated as the number of days from flowering to physiological maturity. The grain filling rate (GFR, mg day^-1^) was estimated as the ratio between grain weight and GFD.

Grain quality analyses were performed at the Quality Laboratory of the International Maize and Wheat Improvement Centre (CIMMYT, Mexico). Test weight (TW, kg hL^-1^) was estimated by the AACC method 55-10. The single kernel character system (SKCS) 4100 equipment (Perten Instruments, Sweden) was used to quantify the grain hardness index (GH). Grain protein content (GP, % at 12.5%moisture basis) was determined using a NIR Systems 6500 machine (Foss, Denmark) with a calibration validated using the Kjeldahl method AACC 46–11A (AACC 2010) and the Dumas method (Leco equipment FP828, Leco Instruments, USA). Before milling, the grain samples were tempered with water according to their hardness and the official AACC method 26-95. The samples were milled into refined flour using a Brabender Quadrumat Senior mill (C.W. Brabender OHG, Germany). Flour yield (FY,%) was measured. In the flour samples, protein (FP, % at 14% moisture basis) was determined by a DA7200 NIR machine (Perten Instruments, Sweden). Overall gluten quality (FS, mL) was determined with the SDS-sedimentation test performed according to (Pena et al., 1990). Dough rheological properties were tested in the mixograph (National Mfg. Co.) to obtain optimum dough mixing time (MT, min) and torque (P, %Torque× min) according to the AACC method 54-40A. Additionally, 60 g flour samples were used in the alveograph (Chopin, France) to measure the tenacity/extensibility ratio (P/L) and elasticity or strength (W, J x 10^-4^) according to the manufacturer’s instructions and the AACC method 54-30A. Finally, bread-making quality was assessed using a direct dough method with 100 g of flour (AACC method 10-09). A volume meter measured the bread loaf volume (LV, mL) by rapeseed displacement. The dough water absorption levels used to run the mixograph, alveograph, and baking tests were calculated according to (Guzmán et al., 2015).

### Statistical analysis

Restricted maximum likelihood (REML) was used to estimate the variance components and to produce the best linear unbiased predictors (BLUPs) for the agronomic and grain quality phenotypic data. The MIXED procedure of the SAS-STAT statistical package (SAS Institute Inc, Cary, NC, USA) was used with year and genotype.

The analysis of variance, box plot, and normal distribution plots was calculated using the mean phenotypic values of three years with PROC MIXED and PROC UNIVARIATE PLOTS procedures with the SAS-STAT statistical package (SAS Institute Inc, Cary, NC, USA) ). The phenotypic and genotypic correlations analysis were performed with two years of phenotypic data in Meta-R software (Alvarado et al., 2019; Balduzzi et al., 2019). Heritability was estimated from variance components as follows:


$$h^{2}=\frac{\sigma_{g}^{2}}{\sigma_{g}^{2}+\frac{\sigma_{ge}^{2}}{e}+\frac{\sigma_{g}^{2}}{re}}$$

Where ${\text{σ}}_{g}^{2}$ is the variance of genotypes, $\sigma_{ge}^{2}$ is the interaction of genotype and year variance, e is the number of years, *σ*_*e*_ is the residual variance, and r is the number of replicates per year (geometric mean r=1).

### Prediction equations for loaf volume

To develop an effective predictive model for a complex phenotyping trait (LV) by simple traits, a multiple linear regression model procedure was applied with the BLUPs for two years. This procedure was performed in R software with the lm package using the basic structure “response variable ~ explanatory variable(s)”. The predicted models were validated with 50% of individuals randomly selected as training and 50% as a testing population with 150 iterations.

### GWAS analysis

The landraces were genotyped with 13177 SNPs using the Illumina Infinium 15K Wheat SNP Array at Trait Genetics GmbH (Gatersleben, Germany). After excluding SNPs with more than 20% missing data and minor allele frequency (MAF) less than 0.05, a total of 10090 SNPs distributed throughout the genome remained. Genome-wide association analyses (GWAS) were performed using the TASSEL V5.2.25 software (Bradbury et al., 2007) following a mixed linear model (MLM) (Yu et al., 2006):


y=Xβ+Zu+e

Where y is the vector of BLUPs, *β* is a vector of SNP marker fixed-effects parameters, u is a vector of random additive effects of inbred lines, X and Z represent matrix, and e is a vector of random residuals. The random genotype effect was estimated as Var(u)=PCA+K ${\text{σ}}_{a}^{2}$ , Where PCA is the principal component analysis matrix with three principal components as a fixed effect and K is n x n matrix of pairwise kinship coefficient as a fixed effect, and ${\text{σ}}_{a}^{2}\;$ is the estimated additive genetic variance (PCA+K model) (Yu et al., 2006). Restricted maximum likelihood estimates of variance components were obtained using the optimum compression level (compressed mixed linear model) and population parameters previously determined (P3D) in TASSEL.

A frequently used threshold was established at –log_10_ P > 3, as previously reported in the literature (Condorelli et al., 2018; Mangini et al., 2018; Sukumaran et al., 2018; Rufo et al., 2021; Wang et al., 2021a). Confidence intervals (CI) for marker-trait associations (MTA) were estimated for each chromosome according to the LD decay reported by (Rufo et al., 2019) using the formula reported by (Chardon et al., 2004).


$$S_{i}^{2}={\left(\frac{CI}{3.92}\right)}^{2}$$

Where CI corresponded with the LD decay for each chromosome. To simplify the MTA information, the associations were grouped into QTL hotspots, defined the density of MTAs along the chromosome and calculated as the QTL overview index (Chardon et al., 2004) for each cM of the genetic map (Wang et al., 2014):


$$U=\frac{\frac{nbQTL}{nbE}}{Total\;length\;of\;map}$$

where nbQTL is the number of QTLs and nbE is the total number of experiments.

The physical positions of SNPs were provided according to wheat genome version 2.1 (IWGSC RefSeq v2.1), available at https://wheat-urgi.versailles.inra.fr/ (Alaux et al., 2018). QTL overview index plot was generated by ClicO software (Cheong et al., 2015). Circular Manhattan plots were generated by the CMplot package in R Software (Yin et al., 2021).

### *In silico* analysis of candidate genes

Identification of candidate genes (CGs) was carried out within the window of ±500 kb of the MTAs within the QTL hotspot peaks. The annotation and function of the candidate genes were performed using the wheat genome browser version 1.0 at https://wheat-urgi.versailles.inra.fr/Seq-Repository/Annotations, and positions of CGs in version 2.1 (IWGSC RefSeq v1.0, v2.1) by integrating the different versions of genome sequence following (Alaux et al., 2018). MTAs with different physical and genetic chromosomes were not considered for the CG search.

### Evaluation of genomic prediction

A cross-validation strategy was used to evaluate the genomic prediction within the landrace collection. Ridge regression best linear unbiased prediction (RR BLUP) (Whittaker et al., 2000; Meuwissen et al., 2001) was used to estimate marker effects and develop the prediction equations for genomic selection. We have performed a cross-validation method to calculate the accuracies of the model using 80% of the population as training and 20% of the population as testing with 150 iterations. The analyses were done in R software using the rrBLUP Package (Endelman, 2011).

## Results

### Phenotypic performance

**Table 1** shows a summary of statistics of the genetic variation of the traits for the two years of phenotyping. Most of the variance of quality and agronomic traits were associated with genotypes. The percentage of variability explained by genotype was the highest for W (82%), whereas the lowest for TW (14%). The maximum variability explained by the GxE interaction and genotype was for FP and GP with 30% and 50%, respectively, whereas for MT and P, GxE had no effect. In the case of LV, 63% variance was explained by genotype and 21% by GxE interaction with no environmental variance, similar to MT, P/L. The influence of the environment was around 20% for GP and FP, GH-3%.

**Table 1:** Trait performance across environments.

Trait	VG	VGE	VE	Mean	SD	CV	h^2^	GS accuracies
GY	0.87	0.22	1.19	5	0.87	17.40	0.56	0.52
TW	0.52	0.05	3.25	77.86	1.28	1.64	0.24	-0.03
TKW	7.26	6.25	10.93	43.73	3.89	8.91	0.46	0.46
GH	73.03	64.12	156.95	42.47	13.83	32.57	0.40	0.16
FY	6.91	0.46	2.83	63.92	1.79	2.80	0.81	0.33
GP	0.68	0.42	0.30	12.88	0.70	5.46	0.66	0.55
FP	0.60	0.36	0.24	10.55	0.67	6.30	0.67	0.53
FS	4.21	0.94	0.51	10.02	2.02	20.15	0.85	0.48
MT	0.39	0.00	0.34	1.94	0.50	25.64	0.70	0.59
P	605.47	5.51	499.08	75.12	19.91	26.50	0.71	0.58
W	6290.46	78.19	1316.66	144.55	46.57	32.21	0.90	0.64
P/L	0.45	0.04	0.31	1.3	0.44	33.45	0.72	0.29
LV	4619.28	1538.95	1157.60	644.01	65.06	10.10	0.78	0.43
GFD	6.30	0.67	5.73	32.47	3.00	9.25	0.67	0.65
GFR	0.03	0.0020	0.06	1.2	0.23	19.38	0.44	0.62

VG, Variance of genotype; VGE, the interaction of genotype and environment variance; VE, variance of residual; SE, Standard error; SD, Standard Deviation; CV, Coefficient of Variation; h^2^ heritability of trait; Trait:, GY, Grain yield (t ha^-1^); TW, Test weight (kg hL^-1^); TKW, Thousand kernel weight (g); GH, Grain hardness; FY, Flour Yield (%); GP, Grain protein (%); FP, Flour Protein (%); FS, Sedimentation test (mL); MT, Mixing time of dough (min); P, Torque peak (%Torque*min); W, Elasticity or strength (J*10^-4^); P/L:, alveograph tenacity/extensibility ratio (P/L); LV, Loaf Volume(mL); GFD, Duration of grain filling (days); GFR, Rate of grain filling (mg day^-1^).

The coefficient of variation (cv) ranged from 33.5% for P/L to 2.8% for FY, with an average of 16.8%. Most traits showed moderate to high heritability (0.66-0.90) except for TW (0.24). The traits with the highest heritability were W (0.90), FS (0.85), and FY (0.81). In contrast, GY, TKW, GFR, GH and TW showed the lowest values below 0.60.

**Figure 1:**
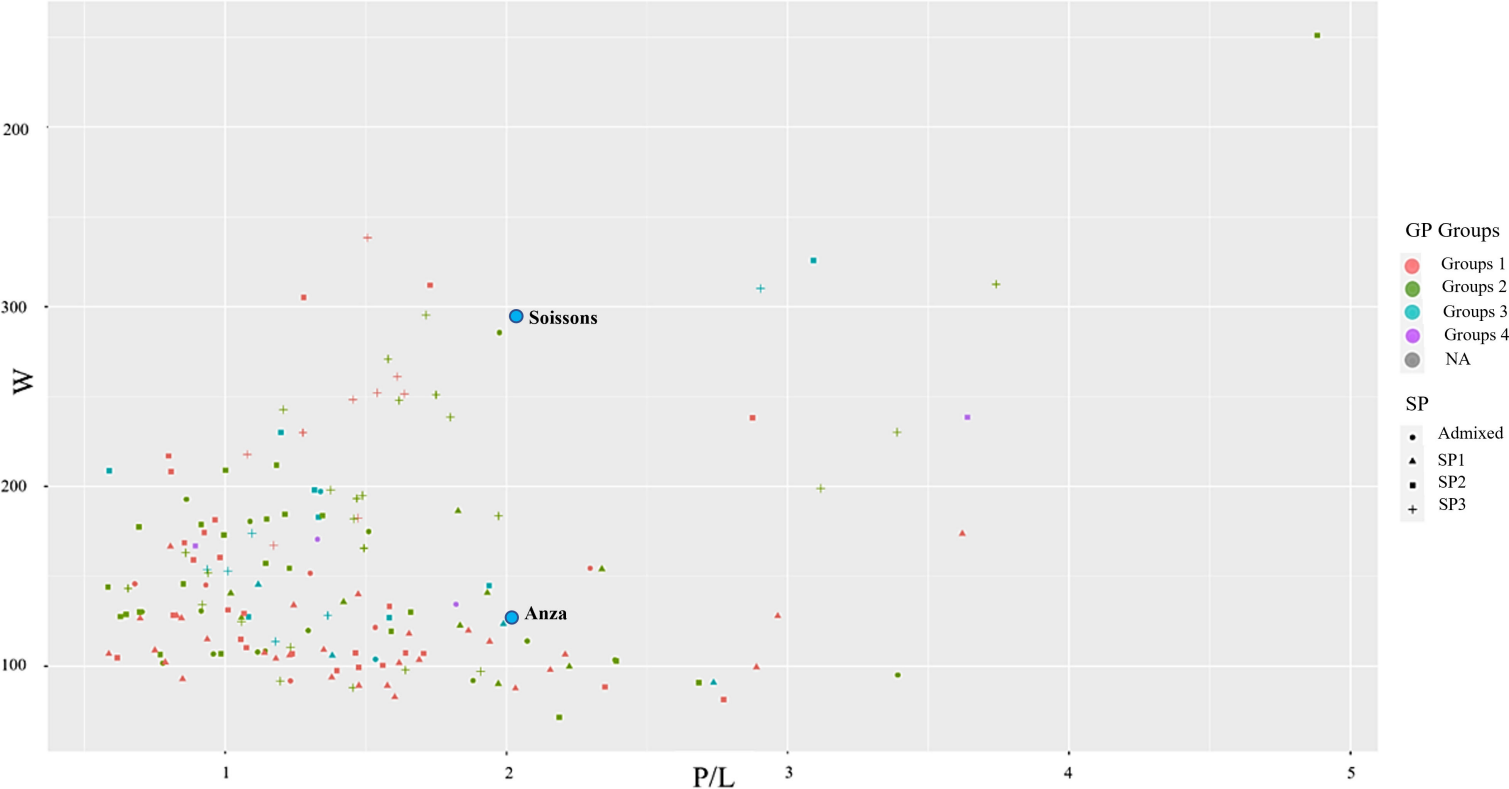
Comparison of grain protein concentration with alveograph traits. Grain protein (GP) is coloured based on the quality levels (Group 1≥13%, Group 2≥12%, group 3≥11% group 4>10%), P/L, alveograph tenacity/extensibility ratio (P/L), W Elasticity or strength (J*10^-4^); NA, not assigned.

To identify Mediterranean landraces as a source for improving grain quality in breeding programs, the genotypes were grouped based on the three quality parameters used nowadays (Pena, 2002) (**Figure 1**). The protein content in grains was classified into Group 1- ≥13%, Group 2- ≥12%, Group 3- ≥11, and Group 4->10. As is shown in **Figure 1**, for the rheological properties, most of the landraces with higher protein concentrations also have high P/L (a non-desired trait) but low gluten strength or W. Some exemptions can be observed, for instance, the landraces of protein group 1 (in red) at the top of the plot have a medium-high W and moderate P/L. For instance, accession T-317 showed medium gluten strength (W=208) and medium-low GP, indicating this accession’s high intrinsic gluten quality.

The checks ‘Anza’ and ‘Soissons’ have been included in the plot to show their position in relation to the landraces. They were included in protein content group 3, thus lower than most landraces. For the gluten properties, both checks showed medium tenacity/extensibility ratio (P/L) (2.02 and 2.08 for Anza and Soissons, respectively), being Soissons one of the genotypes with higher gluten strength (W=346), only below the landrace ‘Bistra’ (W=451).

**Figure 2:**
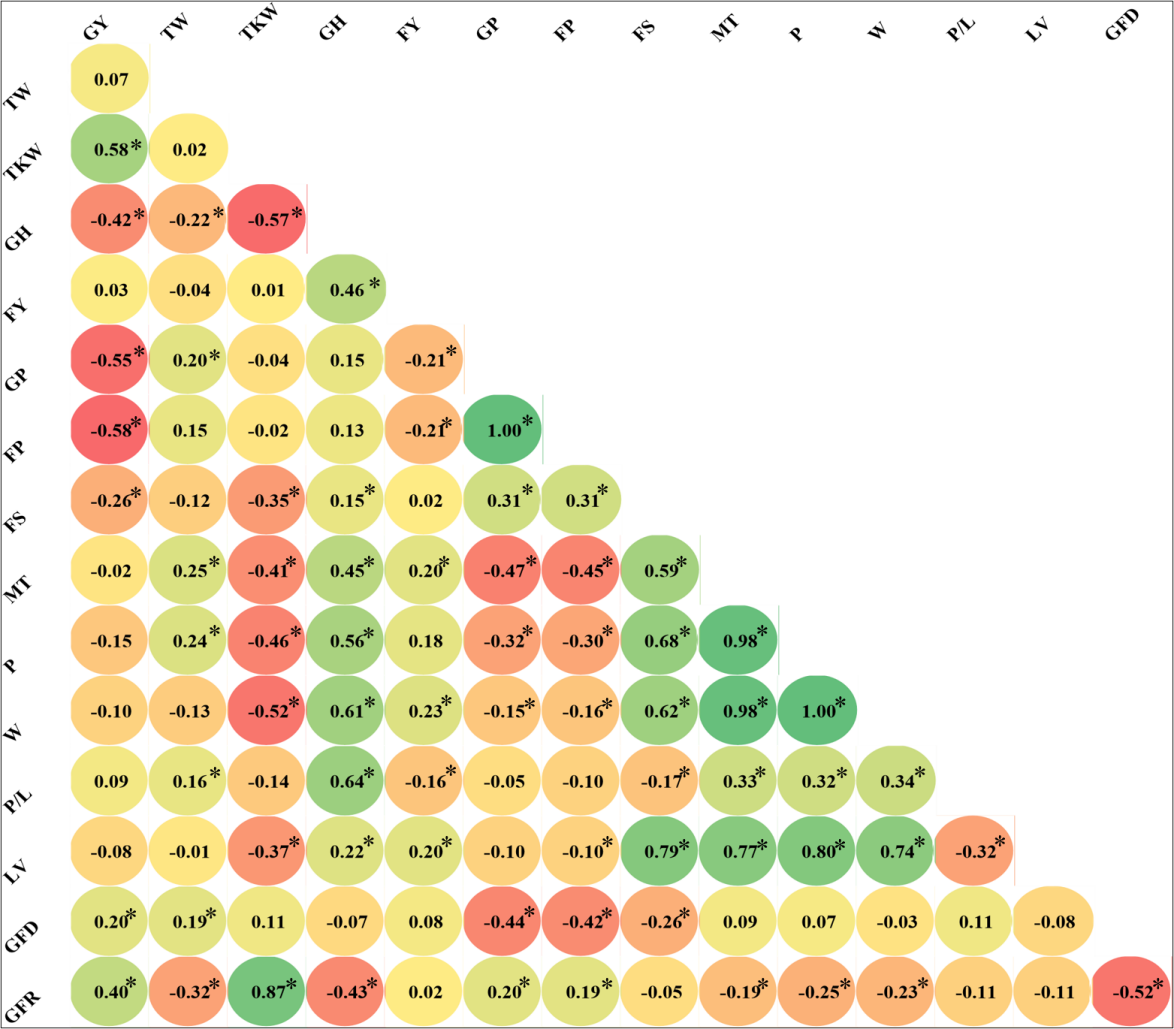
Correlations between bread wheat quality traits measured in a population of Landraces (average of two cropping seasons). Significant Pearson correlation coefficients at p ≤ 0.05 are highlighted with *.Traits, P/L,alveograph tenacity/extensibility ratio; W, Elasticity or strength (J*10^-4^); GFD, Duration of grain filling (days); FP Flour Protein (%); FS, Sedimentation test (mL); FYL, Flour Yield(%); GH, Grain hardness; GP, Grain protein (%); LV, Loaf Volume(mL); MT, Mixing time of dough (min), P, Torque peak (%Torque*min); GFR, Rate of grain filling (mg day^-1^); TKW, Thousand kernel weight (g); TW, Test weight (kg hL^-1^); GY, Grain yield (t ha^-1^)).

The genetic correlations among traits were calculated (**Figure 2**), showing highly significant coefficients between grain and flour protein (1.00). These traits showed, as expected, a moderate but negative correlation with grain yield (-0.55 and -0.58, respectively) and TKW (not significant) and were poorly correlated with FS (0.31), GH (0.15 and 0.13), and TW (0.2 and 0.15). The sedimentation test (FS) positively correlates with the dough’s mixing time, strength, and viscosity. All the rheological properties were highly correlated, excluding alveograph (P/L); the correlation between W, MT, and P was highly significant, as expected, because of the influence of gluten on viscosity properties. As predicted, the GH is also positively correlated with the FY (0.46), MT (0.45), P (0.56), W (0.61), and P/L (0.64). LV correlated significantly with rheological properties (W, 0.74; MT, 0.77; FS, 0.79; P, 0.8). Grain filling duration was negatively correlated with protein concentrations, whereas the grain filling rate (GFR) had a positive and poor correlation. GY, TKW, GFD, and TW were positively correlated. TW and GFD were negatively correlated with the GFR (-0.32 and -0.52, respectively). TKW was negatively correlated with GH (-0.57), FS (-0.35), P (-0.46), MT (0.41), W (-0.52) and LV (-0.37).

When correlations were calculated independently for the different genetic subpopulations (**Supplementary Table 2**) lower number of significant correlations were observed. It is remarkable the difference for TW and FY significant correlations between SP1 and the whole collection, TW and protein content (GP and FP) between SP2 and the whole collection, and finally GY, TW, FY and protein content (GP and FP) between SP3 and the whole collection (**Figure 2** and **Supplementary Table 2**). Differences in significance were observed for different traits between subpopulations. For example GFR was highly correlated with FS, MT, P, W, P/L and LV in SP1 but not in SP2 and SP3. GFR correlated negatively with protein content in SP3, but positively in SP1 and SP2. The opposite situation was between P and W, that were positively correlated in SP3, but negatively in SP1 and SP2.

### Prediction equations for loaf volume

The complexity of the analyses of some quality traits is a bottleneck in screening large germplasm collections. Multiple linear regression equations were developed to predict a complex trait such as loaf volume using a maximum of three simpler traits (**Table 2**). Seven quality parameters were selected from simple to complex phenotyping methods (GP<GH<FY<FS<MT<W<P/L). Initially, the LV was predicted by simple traits (GP+FS+GH) with a multiple regression R^2^ of 0.67 (Equation 1). In equation 2, the prediction ability was increased from 0.67 to 0.73 by replacing GH with MT (a time-consuming measurement of bread wheat quality) (**Table 2**). The multiple regression (Equation 3), including flour mixing time (MT), flour yield (FY), and sedimentation (FS) (R^2 =^ 0.72), was also significant. The combination of simple traits (FS) and complex traits (W+P/L) in the model resulted in the best predictive model with an R^2^ of 0.82. Moreover, including complex traits in the model (Equation 5) did not result in the highest predicting ability compared to Equations 2 and 3. The five models were tested for cross-validation to check the correlation between predicted and observed, and the correlations ranged from 0.74 to 90% (**Table 2**).

**Table 2:** Prediction equations for loaf volume.

Equation	Multiple R-squared	Adjusted R-squared	Residual standard error	P-Value	r^2^
1) LV=GP+FS+GH	0.67	0.66	38.33	< 2.2e-16	0.74
2) LV=GP+FS+MT	0.73	0.73	34.58	< 2.2e-16	0.85
3) LV=MT+FYL+FS	0.72	0.71	34.82	< 2.2e-16	0.84
4) LV=FS+W+P/L	0.82	0.81	28.02	< 2.2e-16	0.90
5) LV=MT+W+P/L	0.75	0.74	32.56	< 2.2e-11	0.80

P/L:, alveograph tenacity/extensibility ratio (P/L); W, Elasticity or strength (J*10^-4^); FS, Sedimentation test (mL); FY, Flour Yield(%); GH, Grain hardness; GP, Grain protein (%); LV, Loaf Volume(mL); MT, Mixing time of dough (min).

### Identification of marker-trait associations and QTL hotspots

**Figure 3:**
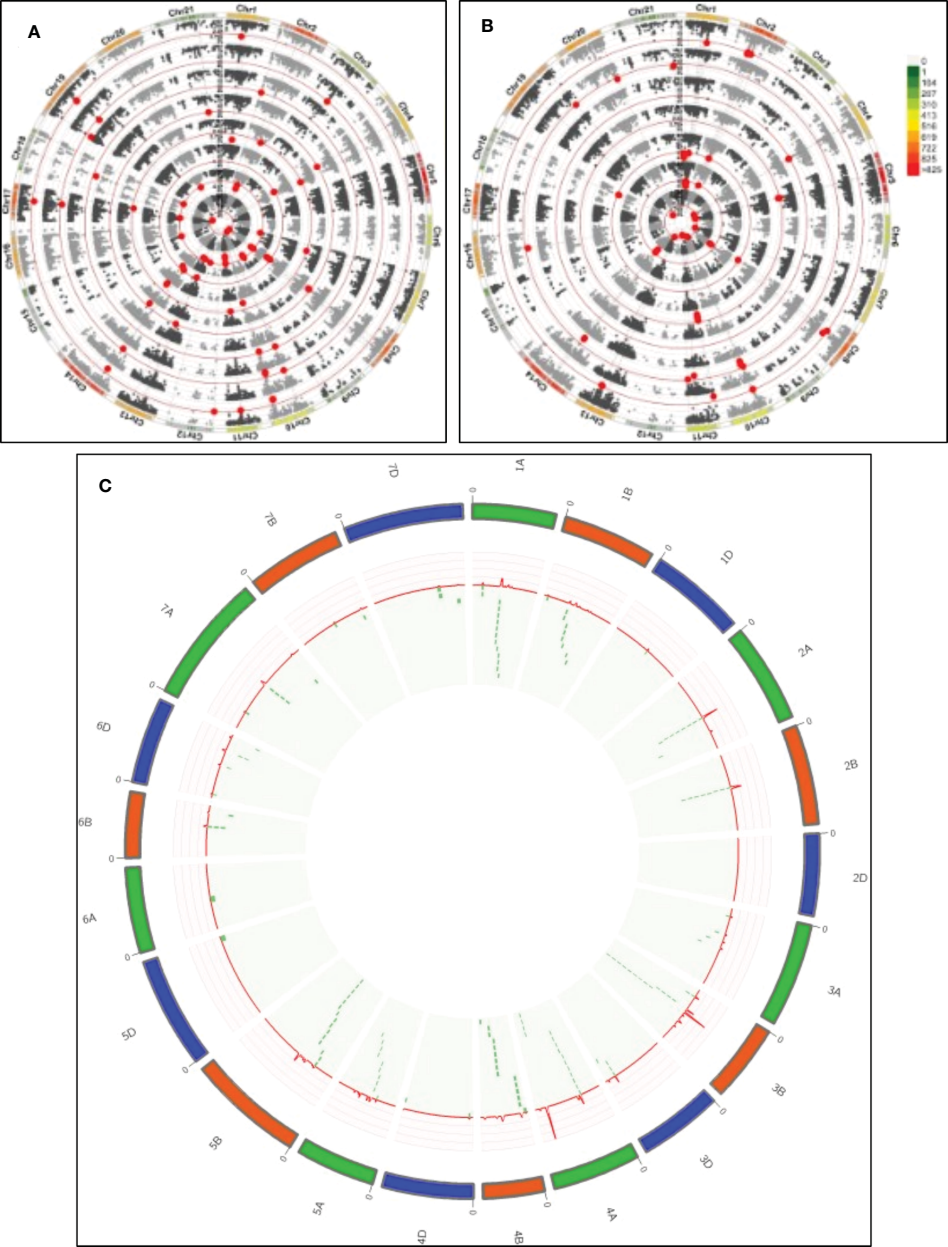
QTL detection for quality and agronomic traits. **(A)** From inside out, GWAS for P/L, W, FP, FS, GP, LV, MT, P. **(B)** From inside out, GWAS for GFD, FY, GFR, TKW, TW, GY, GH. The colour bar indicates the density of SNP markers along the chromosomes. Red dots indicate significant MTAs (-log P>3). **(C)** QTL overview index. The index values (peaks) are represented along chromosomes as a red line. Green dashed tiles represent the significant MTAs below the QTL overview index.

A summary of marker traits associations (MTA) for the mean values of the traits across two years is shown in **Supplementary Table 3** and **Figures 3A, B**. Using a false discovery rate (FDR) approach for detecting spurious associations, only 2 MTAs showed a -log *P*>4.8 at *P*<0.05. Thus, a standard threshold previously reported in several studies in the last years in wheat (Condorelli et al., 2018; Mangini et al., 2018; Sukumaran et al., 2018; Rufo et al., 2021; Wang et al., 2021a) at -log *P*≥3.0 was used. A total of 165 significant MTAs were detected throughout the genome, excluding chromosome 2D (**Figure 3**, **Supplementary Table 3**). The proportion of phenotypic variance of MTAs (R^2^) ranged from 6.66% to 13.02%, with a mean of 8.44% across all the traits (**Supplementary Table 3**). The highest average R^2^ was identified for P (10.17%) and the least for GFR (7.24%). The number of MTAs detected was 71, 78, and 16 for A, B, and D genomes. The highest number of associations were detected on chromosomes 4A (27), 3B (24), 5B (15), and 1A (14). In contrast, chromosomes 1D, 5D, and 6A reported only one MTA. Among 165 MTAs, 25 MTAs were significant for more than one trait, referred to as multi-trait MTAs, and the remaining 140 were trait-specific MTAs. The trait with the highest number of MTAs detected was dough strength (W), with 20 MTAs, showing an allelic effect ranging from 60.52 j×10^-4^ to 68.3 j×10^-4^. In contrast, only 3 trait-specific MTA were detected for P/L and TW. Eleven and fifteen markers were significantly associated with grain and flour protein concentration, respectively, with allelic effects from -0.76% to 0.84%. Among them, nine SNPs were significant for both traits. Thirteen MTAs for MT (R^2^ from7.93% to 11.27%, allelic effect=-0.52 to 0.53 min) and 14 MTAs for PEAK (R^2^ from 7.63% to 12.16%, allelic effect=-21.51 to 25.80%Torque×min) were identified; among them, 11 were in common for both traits, and only 2 were trait-specific for MT, and 3 for P. Flour sedimentation test reported 10 MTAs (6 trait-specific MTAs) and for loaf volume 4 MTAs (3 trait-specific MTAs). Ten and thirteen trait-specific MTAs were identified for FY (R^2^ from 7.27% to 11.35% and allelic effect from -2.45% to 1.85%) and GH (R^2^ from 6.78% to 9.01% and allelic effect from -16.85% to 18.81%) respectively. The allelic effect for all multi-trait MTAs was in the same direction except 3 SNPs (GENE-1350_36, Ra_c22880_760, Ra_c71628_188) on chromosome 2A, which were significant for protein concentration and test weight with an opposite allelic effect between protein concentration and test weight.

For agronomical traits, we identified 14, 12, 10, and 8 MTAs for yield, GFD, TKW, and GFR, respectively. Among them, two markers associated with yield were also associated with MT and GFD. The rest of the MTAs were trait specific. The mean of phenotypic variation explained by marker (PVE) of GY MTAs (8.33%) was similar to the reported by the quality traits, which ranged from 10.17% for P and 7.41% for TW (mean of 8.5% for all of the quality traits). Whereas, GFD and GFR showed lower PVE of 7.74 and 7.24, respectively.

To identify the genomic regions most involved in trait variation, excluding spurious associations, QTL hotspots were considered, and MTAs were grouped following the QTL overview index developed by (Chardon et al., 2004) as previously reported by (Rufo et al., 2021) (**Figure 3C**). A total of 64 peaks were identified using as a threshold the mean of the overview index across the 21 chromosomes (0.05). In contrast, when the high threshold was used (i.e. 5 times the overview index mean) (0.23), a total of 62 peaks were detected. From these last peaks, a total of 53 hotspots were identified (**Table 3**). The highest number of MTAs were detected in QTL_4A.3 (15), including 7 MTAs for MT and P and 1 for LV, followed by QTL_3B.3(13), including 5 MTAs for GP and 8 MTAs for FP.

**Table 3:** Summary of QTL hotspots for quality and agronomic traits.

QTL Hotspot	CI (cM)	MTAs	N traits	Traits
QTL_1A.1	24-26	2	1	GFR
QTL_1A.2	68-72	7	3	FY, P, GFR
QTL_1A.3	78-79	1	1	FY
QTL_1A.4	84-89	3	2	FS, FY
QTL_1A.5	95-96	1	1	GH
QTL_1B.1	9-10	1	1	LV
QTL_1B.2	64-66	2	1	GH
QTL_1B.3	78-81	3	2	GH, W
QTL_1B.4	90-91	1	1	GFR
QTL_1B.5	111-112	1	1	FY
QTL_1B.6	120-121	1	1	FS, FY
QTL_2A.1	103-107	8	4	FP, GFD,GP,TW
QTL_2A.2	143-144	1	1	FS
QTL_2B.1	81-85	8	2	GFD, TKW
QTL_3A.1	66-67	1	1	FS
QTL_3B.1	20-21	1	1	GFR
QTL_3B.2	56-58	4	2	FY, GH
QTL_3B.3	65-67	13	2	FP,GP
QTL_3B.4	74-75	1	1	GH
QTL_3B.5	89-92	3	1	W
QTL_3D.1	129-131	3	1	W
QTL_4A.1	49-53	7	4	GFD, GH, GY, LV
QTL_4A.2	56-59	4	2	GY, MT
QTL_4A.3	136-139	15	3	LV, MT, P
QTL_4B.1	5-6	1	1	GH
QTL_4B.2	14-18	5	1	TKW
QTL_4B.3	60-65	6	3	FS, P, W
QTL_4B.4	74-77	2	2	GFD, GY
QTL_4B.5	103-106	2	1	GY
QTL_4D.1	9-10	1	1	P
QTL_4D.2	170-171	1	1	W
QTL_5A.1	62-65	3	2	GFD, W
QTL_5A.2	70-71	2	2	FP, GP
QTL_5A.3	89-91	2	1	GH
QTL_5A.4	94-95	1	1	GH
QTL_5A.5	104-105	1	1	W
QTL_5B.1	51-53	2	2	MT, P
QTL_5B.2	56-57	1	1	FP
QTL_5B.3	76-79	3	1	GY
QTL_5B.4	82-84	3	1	FY
QTL_5B.5	90-91	1	1	FY
QTL_5B.6	97-100	6	3	FP, FS, GP
QTL_6B.1	70-73	3	3	MT, P, W
QTL_6B.2	105-106	1	1	FS
QTL_6D.1	9-10	1	1	P/L
QTL_6D.2	82-83	1	1	LV
QTL_6D.3	118-119	2	1	GFR
QTL_7A.1	43-44	1	1	MT
QTL_7A.2	125-128	4	3	GFD, MT, P
QTL_7A.3	222-223	1	1	GY
QTL_7B.1	89-90	1	1	W
QTL_7B.2	162-163	1	1	GY
QTL_7D.1	160-163	2	1	GP

P/L:, alveograph tenacity/extensibility ratio (P/L); W, Elasticity or strength (J*10^-4^); GFD, Duration of grain filling (days); FP Flour Protein (%); FS, Sedimentation test (mL); FYL, Flour Yield(%); GH, Grain hardness; GP, Grain protein (%); LV, Loaf Volume(mL); MT, Mixing time of dough (min), P, Torque peak (%Torque*min); GFR, Rate of grain filling (mg day^-1^); TKW, Thousand kernel weight (g); TW, Test weight (kg hL^-1^); GY, Grain yield (t ha^-1^).

The two major QTL hotspots for yield-related traits were QTL_2B.1 with 8 trait-specific MTAs, 5 for TKW and 3 for GFD, QTL_4B.2 with five MTAs for TKW and QTL_5B.3 with 3 MTAs for GY. QTL hotspots, including quality and agronomical traits, were QTL_1A.2, QTL_2A.1, QTL_4A.1, QTL_4A.2, QTL_5A.1, and QTL_7A.2.

### *In silico* candidate genes identification

The significant MTAs within the hotspots were considered for candidate genes (CG) screening. In order to delimitate a short region for searching candidate genes, a window of ±500kb was defined for the significant marker in the central point of the QTL hotspot. A total of 807 candidate genes were found across the 52 QTL hotspots (**Supplementary Table 4**). The number of CGs detected per hotspot ranged from 5 in QTL hotspots, QTL_1B.2 (64 – 66 cM) and QTL_5A.5 (104 – 105 cM) to 54 in QTL_2B.1 (81 – 85 cM).

Search for differentially expressed genes (DEG) was performed using the public expression database at http://www.wheat-expression.com/ (Ramírez-González et al., 2018). DEGs were identified in four tissues: roots, leaves/shoots, spikes, and grains. Among the 807 genes, 254 were expressed in grains, but only 10 were grain-specific (**Supplementary Table 4**). Twenty-nine of the 254 CGs were up-regulated, and one hundred and two were down-regulated during the grain developmental stage (Milk stage< soft dough stage< hard dough stage< dough stage and finally ripening stage); the remaining CGs did not show a clear expression pattern (**Supplementary Table 3**). The ten-grain specific CGs were compared in two tissues, the starchy endosperm and aleurone layer, as reported in (Gillies et al., 2012). Seven CGs were located in hotspots, including quality traits TraesCS2A03G1224100, TraesCS3B03G0177500, TraesCS3B03G1232500, TraesCS4A03G0658300, TraesCS5B03G0662800, TraesCS6D03G0007800, TraesCS7A03G0412300 and remaining three (TraesCS1A03G0034100, TraesCS2B03G0319700, TraesCS2B03G0320000) in hotspots for duration and rate of grain filling (**Table 4**). The CGs from quality hotspots, TraesCS2A03G1224100, TraesCS3B03G0177500, TraesCS7A03G0412300, and TraesCS5B03G0662800, are highly expressed in starchy endosperm compared to aleurone layer. Whereas CGs TraesCS3B03G1232500 and TraesCS4A03G0658300 have higher expression levels in the aleurone layer than in starchy endosperm. Grain-specific CGs for GFR and GFD hotspots are highly expressed in the starchy endosperm compared to the aleurone layer.

**Table 4:** List of genes differentially expressed in the grain.

Hotspots	CI	Trait	Gene ID (v2.1)	Description	tpm starchy endosperm	tpm aleurone layer
QTL_1A.1	24-26	GFR	TraesCS1A03G0034100	Defensin	6.46	2.96
QTL_2A.2	143-144	FS	TraesCS2A03G1224100	Antimicrobial peptide	10.63	3.40
QTL_2B.1	81-85	GFD	TraesCS2B03G0319700	Aspartic proteinase nepenthesin	6.21	1.70
QTL_2B.1	81-85	GFD	TraesCS2B03G0320000	Universal stress protein	0.21	0.32
QTL_3B.2	56-58	GH	TraesCS3B03G0177500	Cysteine proteinase inhibitor	7.49	2.84
QTL_3B.5	89-92	W	TraesCS3B03G1232500	COX3 mRNA-specific translational activator PET494	0.00	6.79
QTL_4A.1	49-53	GH	TraesCS4A03G0658300	Dehydrin	1.51	4.57
QTL_5B.1	51-53	MT, P	TraesCS5B03G0662800	Subtilisin-like protease	4.31	0.09
QTL_6D.1	9-10	P/L	TraesCS6D03G0007800	SKP1-like protein	1.73	0.00
QTL_7A.2	125-128	MT, P	TraesCS7A03G0412300	Protein curvature thylakoid chloroplastic-like	8.46	5.74

tpm, (transcript per million); P/L:, alveograph tenacity/extensibility ratio (P/L); W, Elasticity or strength (J*10^-4^); GFD, Duration of grain filling (days); FS, Sedimentation test (mL); GH, Grain hardness; MT, Mixing time of dough (min), P, Torque peak (%Torque*min); GFR, Rate of grain filling (mg day^-1^).

### Evaluation of genomic prediction

Prediction performance was evaluated following a cross-validation approach using a random selection of 80% of the landraces as training population and 20% as testing population in 150 iterations. The genomic prediction accuracies corresponding to the mean of the 150 iterations are shown in **Table 1**. The results for agronomical traits were 0.65, 0.62, 0.46, and 0.63 for GFD, GFR, TKW, and GY, respectively (**Table 1**). The accuracies for agronomic and quality traits were moderate to high, ranging from 0.65 for GFD to 0.43 for LV. Traits with lower prediction accuracies were FY (0.33), GH (0.16) and TW (-0.03). QQ plots for the accuracies of the iterations followed the normal distribution (**Supplementary Figure 1**).

**Figure 4:**
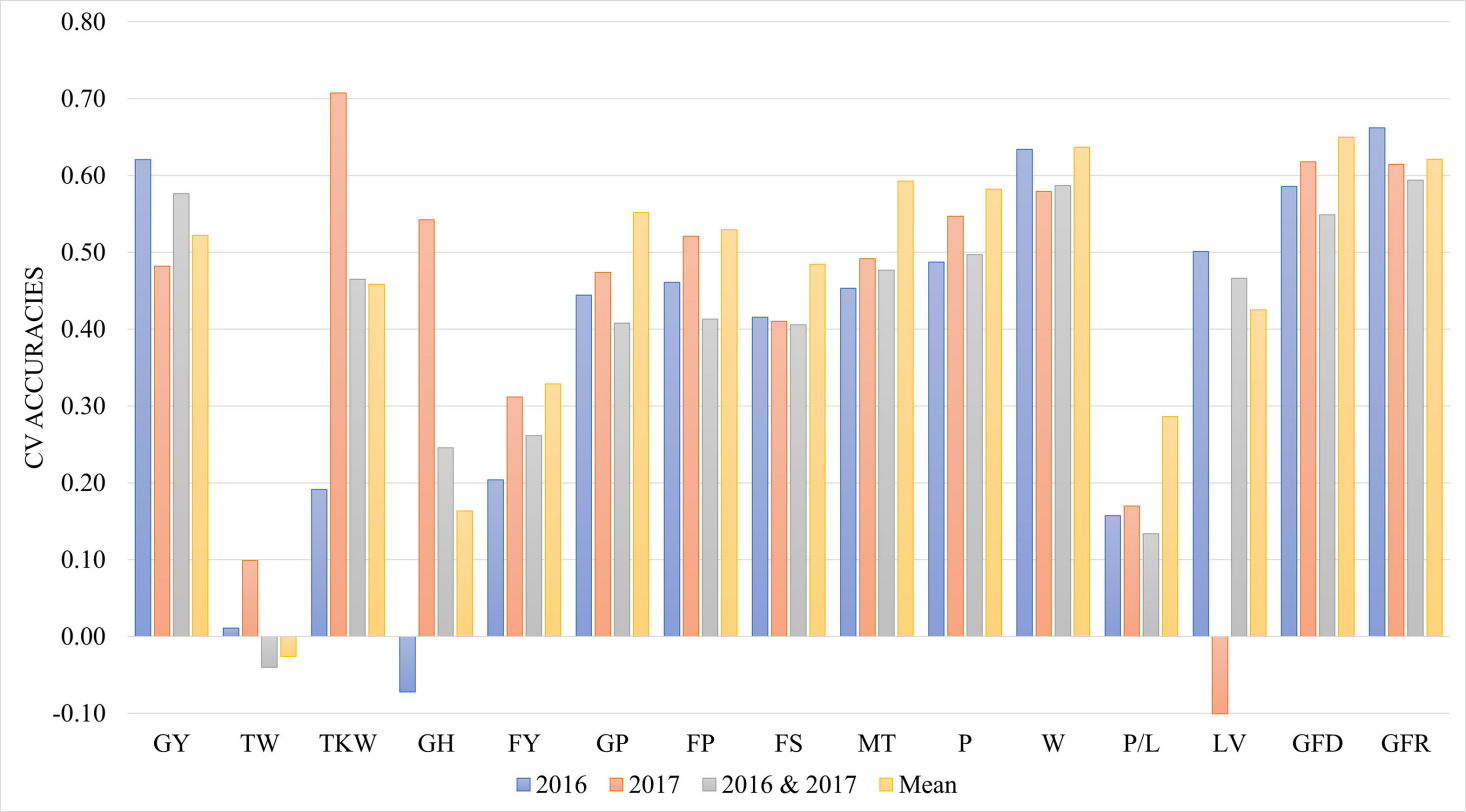
Genomic prediction accuracies using cross validation during 2016, 2017, joint 2016 and 2017 data, and mean across years. CV- Cross Validation; GY- Grain yield (t ha-1); TW, Test weight (kg hL,1); TKW, Thousand kernel weight (g); GH, Grain hardness; FY, Flour Yield (%); GP, Grain protein (%); FP, Flour Protein (%); FS, Sedimentation test (mL); MT, Mixing time of dough (min); P, Torque peak (%Torque*min); W, Elasticity or strength (J*10,4); P/L:, alveograph tenacity/extensibility ratio (P/L); LV, Loaf Volume(mL); GFD, Duration of grain filling (days); GFR, Rate of grain filling (mg day-1).

**Figure 4** shows the comparison of cross validation accuracies between the mean data across years with both years analysed separately and joint data of the two years using the year as environmental factor in the model. Using the mean of the data from the two years resulted in the highest prediction accuracies for most of the traits. Joint data of different years did not increase the power of the prediction.

## Discussion

The chemical composition of grains makes wheat the preferable crop for baked end products. However, to meet the industry requirements, improving the quality traits without reducing grain yield is necessary. Due to the green revolution at the end of the 1960s, breeding was focused on increasing grain yield, which originated in a reduction in the quality parameters of the new cultivars, particularly protein content, which is considered a significant limitation for the baking industry (Goryńska-Goldmann et al., 2021). To enhance the quality characteristics by increasing genetic diversity, the use of wild relatives and landraces is a valuable approach in pre-breeding activities. Landraces are considered a natural reservoir of genetic variation within the species and an invaluable source of new alleles to widen the genetic variability in breeding populations as they were selected during their migration process and are well adapted to their regions of origin (Lopes et al., 2015; Royo et al., 2022). In previous studies with the plant material used in this work, Rufo et al. (2019) and Royo et al. (2022) found a considerable variation in their genetic background and environmental adaptation and herein, analysis of bread wheat quality properties in landraces are explored.

### Exploring genetic diversity and wide phenotypic variation of bread wheat quality properties in landraces

Bread wheat varieties have different and specific quality properties for adaptation to various baking processes, dough handling properties, and many more (Pena, 2002; Guzmán et al., 2016; Guzman et al., 2019). The classification of end-use product properties considers the quality parameters and the selection of cultivars for each purpose. Overall, the landraces studied herein have shown high protein contents and lower W compared to the check line “Soissons”, which confirms previous results obtained by (Sanchez-Garcia et al., 2015), with Spanish landraces producing low-strength flours. Moreover, a wide range of variation was observed for most of the bread wheat quality parameters tested in this important collection of wheat landraces, as shown by the high proportion of variation explained by ‘genotype’ in the analysis of variance. Although environmental factors influenced the quality parameters of bread wheat, the genotype variance stood higher in the landraces compared to the genotype by the environment interaction (Graybosch et al., 1996; Huebner et al., 1997; Zhu and Khan, 2001).

Moreover, the ratio of the variance components of genotype and genotype × environment of protein concentrations and grain hardness compared to milling, mixing time, baking, and rheological traits were low. Therefore, the influence of the genotype by the environment interaction was strong in grain hardness and protein concentration compared to other quality traits. This agrees with Royo et al. (2022), suggesting that human selection to adapt grain quality traits to local preference had more influence than the environment in selecting quality traits.

The landrace population has shown a wide range of variations in kernel hardness. Hard kernels require more energy to crush, and a high amount of water is absorbed due to their coarse texture and high amount of starch damage that is useful for bread making. In contrast, soft kernels require less energy during milling, resulting in a fine flour that is complicated to handle because of less moisture absorption while baking and is used for cakes, cookies, and pastries (Jolly et al., 1993; Pena, 2002; Ikeda et al., 2005). Similarly, grain hardness was significantly correlated with flour yield and rheological properties in this study using landraces. However, the protein and sedimentation tests showed lower Pearson correlation coefficients with grain hardness, which was also observed by Miller et al. (1978).

As expected, the protein concentrations in grain and flour are highly correlated because of the large proportion of protein stored in wheat endosperm. The protein concentrations are negatively correlated with LV and MT, whereas FS is highly correlated due to the gluten strength influence on the bread loaf volume (Færgestad et al., 1999; Uhlen et al., 2004). LV showed a positive correlation with FS and rheological traits (MT, P, W) but was negatively correlated with P/L. It is concluded that the gluten extensibility was directly proportional to the LV in the landraces and these traits respond similarly to previously reported analysis in wheat modern improved germplasm.

Differences in the number of significant correlations between the whole panel of landraces when compared with single subpopulations could be due to the sample size and the statistical power associated to a higher number of genotypes in the analysis that makes it more likely to detect significant differences. However, differences between subpopulations may be due to the genetic differences among them that increase the performance of different traits depending on the environmental conditions. To increase the statistical power of the analysis and make it more likely to detect significant differences between the different genetic backgrounds, to increase the sample size for each subpopulation should be necessary. This could help to reduce the influence of random variation and make it easier to detect any true differences in the relationships between the traits.Phenotype-based selection and prediction of complex traits

Given the ongoing demand of the bakery industry, it is necessary to widen the selection for different traits to cope with the different bakery products (Zhygunov et al., 2020). Hence the raw material with high flour yield, protein content, and strong or weak gluten is essential.

According to the biplot showing the three traits used for quality assessment (**Figure 1**), different landraces can be selected for pre-breeding activities to introgress those traits in the breeding programs or to develop new mapping populations for candidate genes identification. South-western Mediterranean landraces (SP1) were clearly distinguishable from those from northern and eastern regions by their lower value for W (W<200 J×10^-4^), that may be due to the preference in this region for flat breads, which require less gluten strength, as previously suggested by Royo et al. (2022). Accessions Bistra, TRI 8358, TRI 17938, TRI 17006, Pades, and Gemir - B showed all high levels of gluten strength (W>300 J×10^-4^) and could be interesting sources for this trait, although at the same time, their gluten was not extensible (P/L always higher than 1.5). On the other hand, accessions Florence 193, TRI 2100, Isla de Fuerteventura, T-317, Mokhtar, and Candeal were identified as excellent sources of gluten extensibility (P/L> 0.7). However, in this case, all of them, except accession T-317, showed inferior gluten strength. Accession T-317 showed medium gluten strength (W=208 J×10^-4^) having medium-low GP, which indicates this accession’s high intrinsic gluten quality. In terms of end-use quality (bread-making in the case of the current study), accessions Moriborska, Mars Rouge Sans Barbe, and TRI 7821 were the best performers (LV> 770 ml). The three of them exhibited medium gluten strength (W= 216-208 J×10^-4^) combined with balanced gluten (P/L ≤ 1). Of these three accessions, TRI 7821 is probably the most interesting from the breeding point of view, as it performed similarly to the other two accessions but showed almost 1% less protein content, indicating its superior intrinsic gluten quality.

It is also of special interest the prediction of complex traits by the most straightforward traits to reduce phenotyping efforts (Lindhauer, 2012). Loaf volume is a key trait in the baking industry. However, phenotyping loaf volume is expensive, time-consuming and requires well-equipped laboratories with specialized technicians to evaluate in an extensive breeding program population. The dough end-use quality tests also needed a large quantity of flour. Therefore, the trend of indirect selection by predicting the baking quality traits became common practice using obligatory quality indicators like protein content and alveograph traits that are evaluated in the selection of wheat varieties (Lindhauer, 2012).

The results of multiple linear regression to predict loaf volume by multiple traits depending on the complexity of phenotyping concludes that the prediction ability is increased by using simple traits to complex traits. Model equation 2 with manageable traits was intermediate compared to the complexity of traits in equations 3 and 5; equation 4 stood outstanding by using complex traits. Similar models were previously performed using modern cultivars (Rózylo and Laskowski, 2011; Gabriel et al., 2017; Laidig et al., 2018), and this is the first time that these models are also reported in a wheat landrace population of non-improved germplasm.

### Marker trait associations

Dissecting the genetic bases of complex traits in crop breeding is essential to carry out molecular-based approaches to improve cultivars. Numerous attempts have been previously done to identify QTLs and marker–trait associations linked to traits of interest to achieve marker-assisted selection (MAS) approaches and the introgression of alleles of commercial interest in improved genotypes (Pandurangan et al., 2022).

The distribution of the 165 MTAs across genomes is similar to the reported by (Rufo et al., 2021) using agronomic and vegetation indices, 43%, 47% and 10% for genomes A, B and D, respectively. Other studies with similar results (Chao et al., 2010; Wang et al., 2014; Gao et al., 2015) explained this distribution of MTAs in the 3 genomes as a consequence of the lower genetic diversity and higher linkage disequilibrium found in the D genome in comparison with A and B genomes.

To reduce the complexity of the high number of MTAs found by the GWAS analysis, QTL hotspots were defined using the QTL overview index developed to identify the regions of the genome with a higher density of QTL peaks (Chardon et al., 2004). The suitability of this approach is reflected in the selected hotspot regions, as they reported a higher number of MTA density (**Figure 3C**). Comparison with other studies mapping quality traits in the last year showed the presence of common genomic regions among Mediterranean landraces and other germplasm types. Four regions co-localized with the study from (Battenfield et al., 2018) using CIMMYT breeding lines (1A.2, 1B.2, 4B.2, and 7A.2), although these common QTL regions showed associations for different traits. Two QTL hotspots (3B.5 and 4B.5) were in similar positions to those reported by Yang et al. (2019) in a Chinese winter wheat collection, but they did not share common traits. The study reported by (Hao et al., 2022) also with a collection of Chinese breeding lines that shared three regions, 2B.1, 3D.1, and 4B.5, although the last one reported an association with yield in our study. Finally, when comparing our results with (Rathan et al., 2022), using a diverse panel including breeding lines, landraces, and synthetic lines, four common regions were observed: 1A.2, 3B.3, 3B.5, and 5B.1. Only in the hotspot 3B.3 associations for the same traits were found (protein content). Identifying common genome regions among different studies but mapping different traits may indicate the complexity of the analysed traits, clustering genes controlling different quality traits or genes showing pleiotropic effect. Identifying them could be of interest to pyramiding different genes in future breeding programs. Additionally, using the reference genome makes possible a rapid identification of common molecular markers among the different studies that can be used for marker-assisted breeding.

### *In silico* analysis of candidate genes

Using the gene annotation from the ‘Chinese spring’ reference genome sequence (IWGSC, 2018) led to the identification of 807 gene models within the 53 QTL hotspots. CG mining was carried out by searching for grain-specific DEGs in different tissues through in-silico analysis at http://www.wheat-expression.com.

Our results indicate that 10 candidate genes were specifically expressed in grain. Among them, those found in the GFR and GFD hotspots were mainly expressed in starchy endosperm. The CG TraesCS3B03G0177500 located in the hotspot 3B CI of MTA significant for GH is also mainly expressed in the starchy endosperm. This CG encodes a cysteine proteinase inhibitor protein. This dominant protein plays a crucial role in the transportation of storage proteins and is also involved in the hydrolysis of gluten protein responsible for the rheological properties of dough in wheat (Koehler and Ho, 1990; Capocchi et al., 2000; Mikola et al., 2001; Mäkinen and Arendt, 2012). As reported by (Usman et al., 2021), a mutation in the gene encoding Cysteine proteinase inhibitor protein in rice resulted in a positive regulation during grain development and increased grain yield (Usman et al., 2021).

Other CG expressed in different tissues were found by other authors to be involved in quality traits. The SNP, Ra_c71628_188 within the QTL hotspot, QTL_2A.1 is present within the CG TraesCS2A03G0724700 encoding for an alpha amylase that is an important enzyme involved in the degradation of starch (Majzlova et al., 2013). The overexpression of a known isoform of AMY3 resulted in a high level of alpha-amylase activity in harvested grains, producing an increase in loaf volume when baking additives were added in the baking process (Ral et al., 2016). Multi trait MTA SNP Ra_c22880_760 in QTL_2A.1 is in the coding region of CG TraesCS2A03G0724000 encoding a potassium transporter. As reported by (Lu et al., 2014) the higher accumulation of potassium also significantly increased kernel size and weight and a positive change in the glutenin/gliadin ratio concentration was observed. The multi-trait MTA wsnp_Ku_c3185_5949143 is significant for MT, W, and P and is located in the coding region of TraesCS6B03G0922000 involved in ADP ribosylation. ADP-ribosilation factors (Arf) proteins are involved in avoiding or clearing the vesicles trafficking that occurred during the transportation of gluten proteins from the rough endoplasmic reticulum (ER) to storage vacuoles *via* the Golgi complex and vesicles in ER (Wennerberg et al., 2005). Altering this gene led to a gluten strength and protein content change. In addition, the gluten macropolymer and glutenin subunit are affected, resulting in weak gluten strength and extensibility due to the change in the vesicle trafficking (Tyler et al., 2015).

The CG TraesCS4A03G1111500 encodes for a phosphoglucan/water dikinase present in the QTL hotspot QTL_4A.3 involved in MT, P and LV. The SNP RFL_Contig3841_2409 significant for MT and P is present within the CG. To catalyze the phosphorylation of starch, two isoforms of glucan water dikinase are involved; Glucan water kinase1 (GWD1) and phospoglucan water dikinase (PWD1/GWD3) (Edner et al., 2007). Transgenic wheat developed by reducing the expression level specifically in the endosperm, reduced starch phosphate content and increased grain size, enhancing by 29% the grain yield and by 26% biomass (Ral et al., 2012). The difference in the rheological properties and dough preparation in bread wheat differs by the starch content in the flour (Cao et al., 2019; Gao et al., 2020). Similarly, TraesCS1B03G0980800 in QTL hotspot QTL_1B.4 encoded an alpha-glucan water dikinase (GWD), also involved in starch degradation. As reported by (Wang et al., 2021b) the overexpression of GWD1 in rice enhanced the grain yield and quality traits

CGs within the QTL hotspots involved in agronomical traits are mostly related to biotic and abiotic stress resilience, growth regulators, photosynthesis process, and other metabolic processes. For instance, eighteen Peroxidase (POD) and two flowering-promoting genes are detected in the region of QTL_2B.1 responsible for grain filling and grain weight duration. UDP-Glycosyltransferases have been correlated in wheat with the increase of grain wheat in chromosome groups 2 and 7 (Hou et al., 2014). In our study, we detected UDP-Glycosyltransferase genes in chromosomes 1A, 1B, 2B, 3B, 4B, 5A, and 6D, most of them were detected in hotspots involved in yield and yield-related traits. The CG TraesCS1A03G0031500 found in QTL_1A.1 encodes a transforming growth factor beta receptor. The mutation of this gene in rice increased the yield by 15-44% in a greenhouse experiment (Wu et al., 2008). Further experiments are required to evaluate its effect in wheat.

### Genomic prediction within the landrace collection

Genomic prediction or selection (GS) refers to a marker-based selection aimed to enhance the genetic gain of quantitative traits in breeding programs. We used a cross-validation design to evaluate the accuracy of GS in Mediterranean bread wheat landraces to improve quality traits.

The quality traits providing the highest prediction accuracies using mean data of the two years experiments were W, MT and P. In Moderate prediction, accuracies were found in the landrace panel (0.33 – 065) except for TW, GH, and P/L, which reported low accuracies. The low accuracy in TW and GH was expected of its low heritability. The magnitude of the accuracies of quality traits is similar to a previous study in wheat using F_5_-derived F_7_ lines from the CIMMYT wheat breeding program (Battenfield et al., 2016). These results indicated the potential to use genomic selection not only in modern wheat family selection but also when using landraces to increase genetic diversity.

When data of both years of experiments were included in the prediction equation and including the year as environmental factor, the cross validation accuracy for the majority of the traits were relatively high, especially for GY, W, GFD and GFR, which have accuracy values greater than 0.55. Most of the quality traits with accuracies from 0.4 were not influenced by the environment and the mean values showed the highest accuracies. Differences among years were observed specially for traits as GY, TKW, GH and LV, indicating the high influence of environmental conditions in the prediction of these traits. However new experiments under different conditions would be necessary to improve the accuracy of predictions.

## Conclusions

Traditionally, grain yield in wheat has been negatively linked to bread quality properties in diverse sets of wheat germplasm, particularly in modern wheat varieties. However, ideally, farmers and the industry demand quantity and quality of wheat products, and selection methods to build allelic preference for high yield together with high quality are necessary. Herein, genetic variation for wheat quality traits is explored in a population of landraces and significant marker-trait associations were identified in most of the chromosomes.

The baking industries prefer rapid and straightforward procedures for preparing the dough on a large scale. Knowing the genetic control of grain components compared to agronomical characteristics can be an elective way to develop a new variety for commercial purposes. Although the bread wheat quality parameters are highly polygenic, their high heritability indicates that these traits can be easily manipulated in the selection process but considering that their negative correlation with yield can indirectly affect agronomical traits. The genomic regions proposed are potential targets for developing a new allelic variation through genome editing. The landraces used in our study can be useful as a resource for a new natural variation and can be introduced in the breeding programme as a parent.

## Data availability statement

The original contributions presented in the study are included in the article/**Supplementary Material**. Further inquiries can be directed to the corresponding author.

## Author contributions

Conceptualization: JS. Methodology: VY, ML, CG, JS. Formal analysis: VY, JS. Data curation: VY, ML, JS. Writing—original draft preparation: VY. Writing—review and editing: ML, CG, JS. Supervision: ML, JS. Project administration: ML, JS. Funding acquisition: ML, JS. All authors contributed to the article and approved the submitted version.
