# Supplementary material for: Uncovering the genetic basis for quality traits in the Mediterranean old wheat germplasm and phenotypic and genomic prediction assessment by cross-validation test
Source: Front Plant Sci. 2023 Jan 27;14:1127357. doi: 10.3389/fpls.2023.1127357 (PMC9911887; doi:10.3389/fpls.2023.1127357)
Supplement: Supplementary file 1 [file Table_1.docx]

Supplementary Material

Uncovering the genetic basis for quality traits in the Mediterranean old wheat germplasm and genomic prediction assessment by cross validation test

**Venkata Rami Reddy Yannam^1^, Marta Lopes^1^, Carlos Guzman^2^, Jose Miguel Soriano^1^***

*** Correspondence:** Jose Miguel Soriano: josemiguel.soriano@irta.cat

# Supplementary Figures


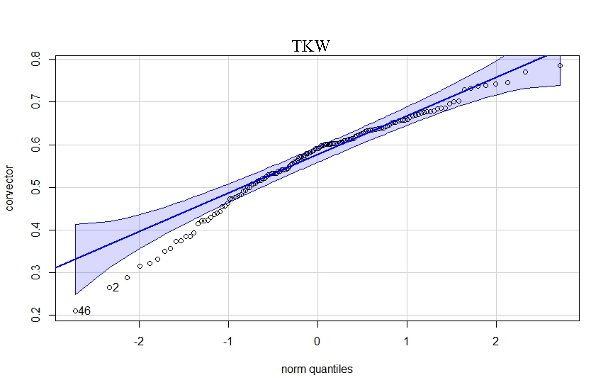

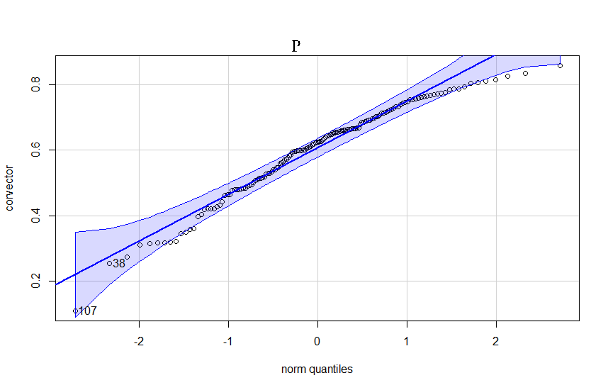

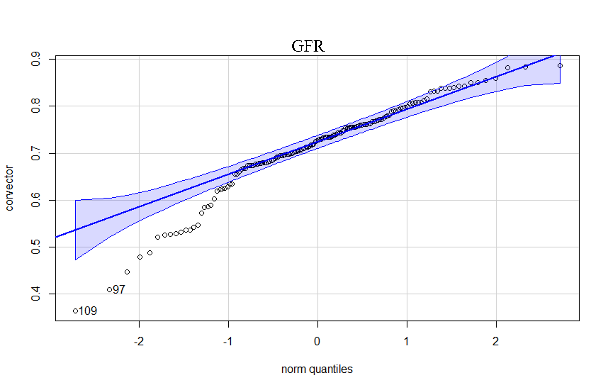

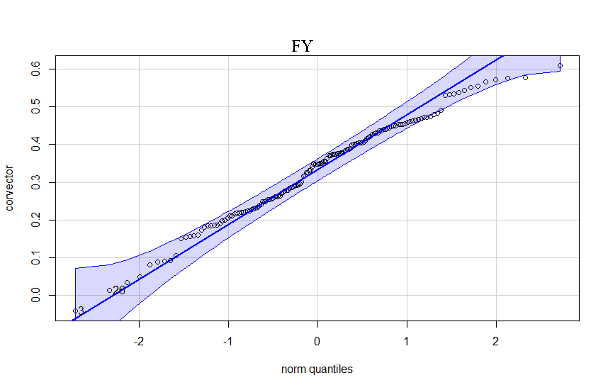

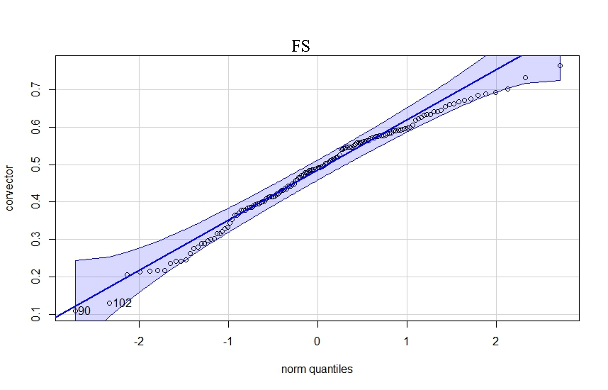

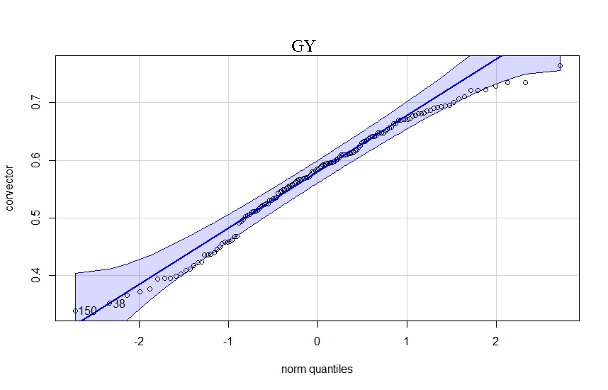

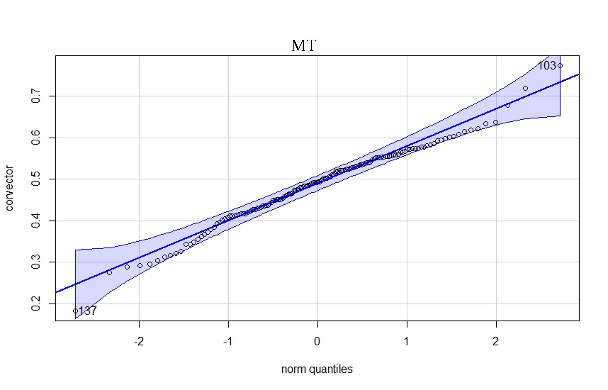

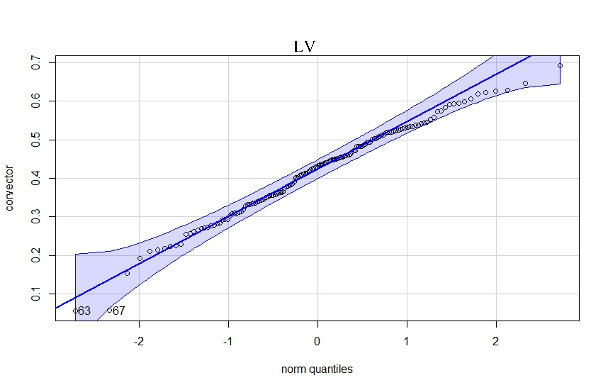

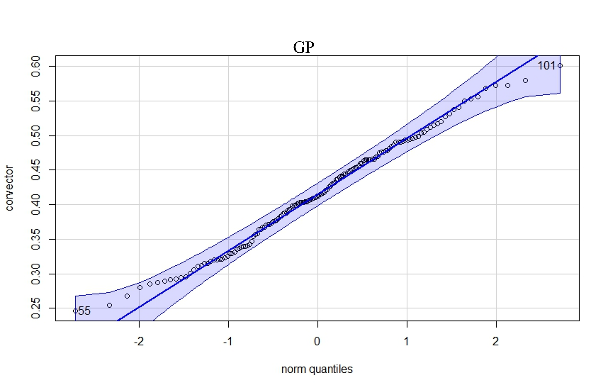

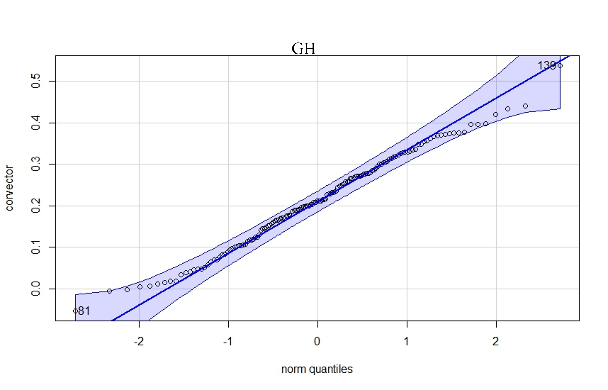

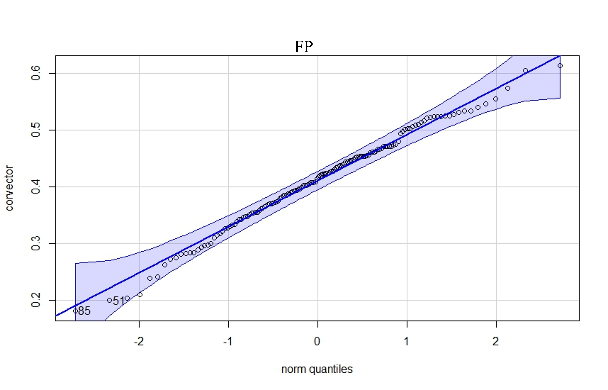

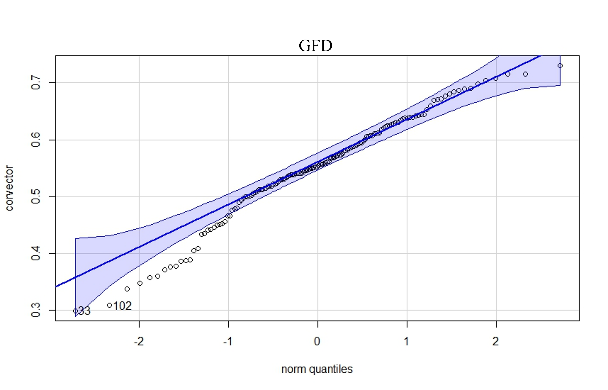

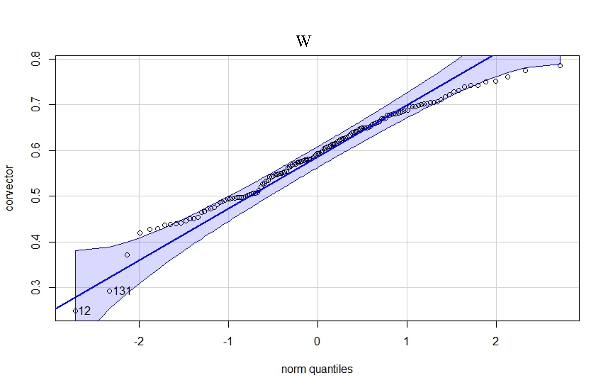

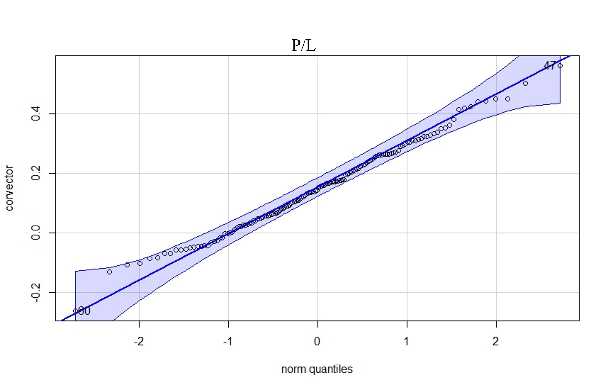

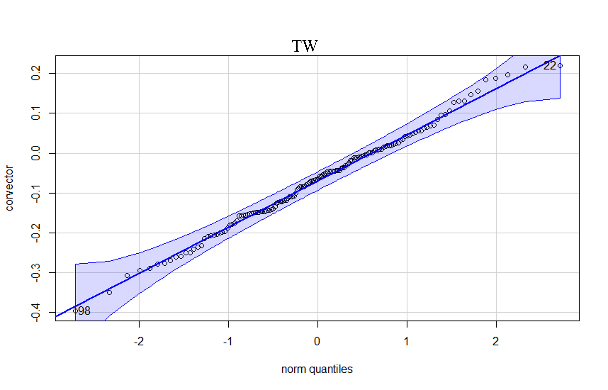


Supplementary Figure 1. QQ plots for cross validation test in the landraces *. P/L-alveograph tenacity/extensibility ratio; W- Elasticity or strength (J*10^-4^); GFD- Duration of grain filling (days); FP Flour Protein (%); FS- Sedimentation test (mL); FYL- Flour Yield(%); GH- Grain hardness; GP- Grain protein (%); LV- Loaf Volume(mL); MT- Mixing time of dough (min), P- Torque peak (%Torque*min); GFR- Rate of grain filling (mg day^-1^); TKW- Thousand kernel weight (g); TW- Test weight (kg hL^-1^); GY- Grain yield (t ha^-1^).
